# Role of the Gut Microbiota in Osteoarthritis, Rheumatoid Arthritis, and Spondylarthritis: An Update on the Gut–Joint Axis

**DOI:** 10.3390/ijms25063242

**Published:** 2024-03-13

**Authors:** Umile Giuseppe Longo, Alberto Lalli, Benedetta Bandini, Roberto de Sire, Silvia Angeletti, Sebastien Lustig, Antonio Ammendolia, Nicolaas Cyrillus Budhiparama, Alessandro de Sire

**Affiliations:** 1 Fondazione Policlinico Universitario Campus Bio-Medico, Via Alvaro del Portillo, 200, 00128 Roma, Italy; g.longo@policlinicocampus.it (U.G.L.); albertolalli30@gmail.com (A.L.); benedettabandini.000@gmail.com (B.B.); 2 Research Unit of Orthopaedic and Trauma Surgery, Department of Medicine and Surgery, Università Campus Bio-Medico di Roma, Via Alvaro del Portillo, 21, 00128 Roma, Italy; 3 Gastroenterology, Endoscopy Unit, IRCCS Humanitas Research Hospital, 20089 Rozzano, Italy; roberto.desire@libero.it; 4 Gastroenterology Unit, Department of Clinical Medicine and Surgery, University Federico II of Naples, 80126 Naples, Italy; 5 Unit of Clinical Laboratory Science, University Campus Bio-Medico of Rome, 00128 Rome, Italy; s.angeletti@unicampus.it; 6 Orthopaedic Department, Lyon North University Hospital, Hôpital de La Croix Rousse, Hospices Civils de Lyon, 103 Grande Rue de la Croix Rousse, 69004 Lyon, France; sebastien.lustig@gmail.com; 7 Department of Medical and Surgical Sciences, University of Catanzaro “Magna Graecia”, 88100 Catanzaro, Italy; ammendolia@unicz.it; 8 Research Center on Musculoskeletal Health, MusculoSkeletalHealth@UMG, University of Catanzaro “Magna Graecia”, 88100 Catanzaro, Italy; 9 Department of Orthopaedics, Leiden University Medical Center, 2333 Leiden, The Netherlands; n.c.budhiparama@gmail.com

**Keywords:** microbiota, gut microbiota, osteoarthritis, rheumatoid arthritis, spondylarthritis, rehabilitation

## Abstract

Dysregulation of the gut microbiota and their metabolites is involved in the pathogenic process of intestinal diseases, and several pieces of evidence within the current literature have also highlighted a possible connection between the gut microbiota and the unfolding of inflammatory pathologies of the joints. This dysregulation is defined as the “gut-joint axis” and is based on the joint–gut interaction. It is widely recognized that the microbiota of the gut produce a variety of compounds, including enzymes, short-chain fatty acids, and metabolites. As a consequence, these proinflammatory compounds that bacteria produce, such as that of lipopolysaccharide, move from the “leaky gut” to the bloodstream, thereby leading to systemic inflammation which then reaches the joints, with consequences such as osteoarthritis, rheumatoid arthritis, and spondylarthritis. In this state-of-the-art research, the authors describe the connections between gut dysbiosis and osteoarthritis, rheumatoid arthritis, and spondylarthritis. Moreover, the diagnostic tools, outcome measures, and treatment options are elucidated. There is accumulating proof suggesting that the microbiota of the gut play an important part not only in immune-mediated, metabolic, and neurological illnesses but also in inflammatory joints. According to the authors, future studies should concentrate on developing innovative microbiota-targeted treatments and their effects on joint pathology as well as on organizing screening protocols to predict the onset of inflammatory joint disease based on gut dysbiosis.

## 1. Introduction

Recently, many research studies have revealed that disruptions in the gastrointestinal microbiota and their byproducts are not merely associated with the pathogenic processes of gastrointestinal illnesses such as IBD and colon cancer. Rather, these disruptions can also trigger several extraintestinal immune and metabolic diseases through a disorder of intestinal homeostasis [1].

Specifically, several pieces of evidence within the current literature highlight a possible connection between the gut microbiota and the development of inflammatory diseases of the joints [2,3,4]. Indeed, a paper in 2017 indicated that lipopolysaccharide, a structural element of Gram-negative bacteria resident in the gut, apparently activates cytokine cascade and causes T-cell-mediated pathogenesis of arthritis [5].

Another study published in 2019 examined the gut microbiome profile of female rheumatoid arthritis (RA) patients, demonstrating dysbiosis and substantial variations in the microbial composition of several taxa among healthy controls and patients. The phylum Bacteroidetes was concentrated in early RA patients, while Actinobacteria, especially the species Collinsella, were enhanced in healthy individuals [6].

This dysregulation was recently identified as the “gut-joint axis” [7]. The mechanisms by which dysbiosis triggers arthritis includes molecular mimicry, inflammatory responses, and the loss of intestinal mucosal integrity [8].

The “gut-joint” axis has its fundaments from the interaction between the gut and joints. It is widely recognized that the gut microbiota produces a variety of compounds, including short-chain fatty acids, enzymes, and metabolites [9]. Consequently, these proinflammatory compounds that bacteria produce, such as lipopolysaccharide, move from the “leaky gut” to the bloodstream leading to systemic inflammation which then reaches the joints, with consequences such as osteoarthritis (OA), rheumatoid arthritis, and spondylarthritis (SpA) [10,11].

These collections of microorganisms lead to a state of dysbiosis, a phenomenon that can be seen simultaneously in both RA and IBD patients [12]. In fact, the composition of the gut microbiome includes components that can be influenced by hereditary factors up to 30%. The cause for this phenomenon might be attributed to the presence of antigens derived from pathogens that exhibit sequence similarity to that of self-peptides. This resemblance could potentially activate autoreactive T or B cells, thereby initiating autoimmunity through a mechanism known as molecular mimicry. This process has been associated with Firmicutes and Proteobacteria and may exert a more significant impact on disease susceptibility in individuals with specific genetic predispositions [13].

According to the literature, patients with inherent genetic and epidemiologic risk factors may experience the initiation of localized autoimmunity at the surface of mucosal tissues due to these environmental triggers. This localized autoimmunity can subsequently extend to systemic autoimmunity, eventually leading to clinically apparent disease [14]. 

Through this comprehensive review, our objective was to summarize the state-of-the-art research in the literature focused on the connections between gut dysbiosis, OA, RA, and SpA, elucidating the diagnostic tools, the main outcome measures, and the potential treatment options for these subjects.

## 2. Gut Microbiota and Arthritis

### 2.1. Microbiota in Osteoarthritis (OA)

More than 25% of people over the age of 18 suffer from OA, which is the most common degenerative joint disease [15].

Pathological modifications found in OA joints include the gradual deterioration and loss of cartilage in the articulation, the thickening of the subchondral bone, the production of osteophytes, different degrees of synovial inflammation, the destruction of the knee ligaments and menisci, and joint capsule hypertrophy [16].

For a long time, OA was thought to be a cartilage degeneration disease. However, our fundamental knowledge of the underlying processes of OA has shifted in the last decade. OA is no longer considered to be a common degenerative disease arising from regular bodily deterioration but rather a complex illness, in which chronic inflammation represents a significant action [16,17].

With an aging and obese population, the frequency of OA has increased, affecting 303 million people all over the world in 2017 and leading to negative humane, clinical, and economic burdens. In addition, the age of incidence, prevalence, and time spent with suffering OA have been increasing in the last years with a negative impact also in terms of pain sensitization [15,18]. Moreover, according to emerging research, the gastric microbiota triggers an inflammatory condition. The proposed concept initiates with the dysregulation of the typical gut balance, followed by continual shifts in the microbial structure and genetic adaptability to facilitate the optimal bacterial accommodation to the host environment. This is coupled with both adaptive and innate immune reactions, which are induced by the migration of bacteria and their derivatives into the bloodstream directed towards the joint [16].

**Figure 1:**
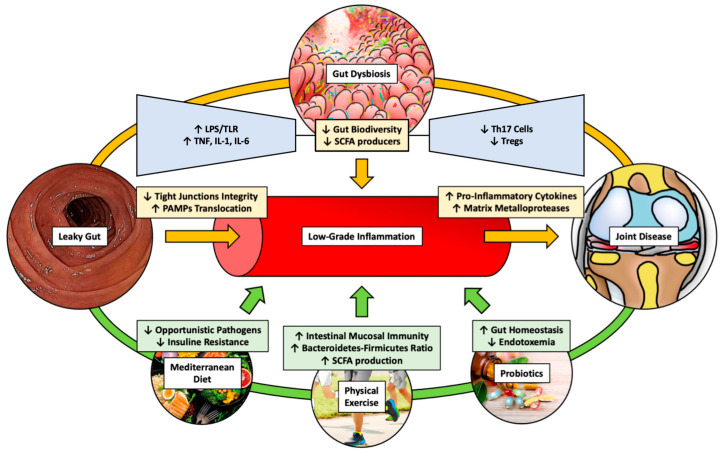
Factors acting on the “Gut-Joint Axis”.

In OA patients, the gut bacteriome demonstrates elevated levels of Actinobacteriota and Proteobacteria along with reduced levels of Firmicutes compared to those of healthy individuals [19]. This cycle eventually leads to joint low-grade inflammation, contributing to the etiology of OA [20]. Figure 1 describes the “gut-joint axis”.

### 2.2. Microbiota in Rheumatoid Arthritis (RA)

RA is a chronic, polyarticular and symmetric inflammatory joint disease characterized by ongoing synovitis that damages cartilage in the articulation and juxta-articular bone [21,22]. Patients usually report discomfort and swelling in the joints of their hands and feet [23]. This swelling typically affects the wrists, metacarpophalangeal, metatarsophalangeal, and proximal interphalangeal joints [24].

A growing body of data from epidemiological and translational studies suggests that interactions between mucosal locations and dysbiotic microbiota may have a causative role in the development of RA. The pathophysiology of rheumatoid arthritis may start in mucosal locations and subsequently progress to the synovial joints [25].

Significant data have been published in recent years demonstrating the presence of changes in the structure of the microbial flora of patients in the preclinical stages of rheumatoid arthritis, implying the importance of intestinal dysbiosis for the onset of RA and the persistence of systemic inflammation [25]. Several studies have shown the presence of DNA from various bacterial species such as Prevotella, Fusobacterium, Porphyromonas, and Bacteroides in the serum and synovial fluids of individuals with RA [13,26,27].

The pathophysiology of the gut–joint axis is still unclear. As a result, gut microbial metabolites may operate as a link to the gut–joint axis in RA, control gastrointestinal barrier and immune homeostasis, and engage in the metabolism of bones [28].

### 2.3. Microbiota in Spondylarthritis (SpA)

SpA comprises a group of inflammatory pathologies that involve spinal and peripheral joint arthritis, inflammation of the junctions of ligaments and tendons to bones, and systemic extra-articular symptoms [29]. SpA is now divided into different subtypes that are namely as follows: psoriatic arthritis, reactive arthritis, arthritis related to IBD, a subgroup of juvenile idiopathic arthritis, and ankylosing spondylitis [10].

Gut dysbiosis is linked with arthritis susceptibility through a variety of routes, according to research. Although there is no consensus regarding the bacterial species involved in the pathogenesis, an enrichment for enteropathogens such as Salmonella, Shigella and Campylobacter has been found in SpA patients [30].

The process that has been recognized for establishing a gut–joint axis through the link between the microbiota of the gut and the host immune system involving shifts in the gut barrier function, molecular mimicry, modifications and disparities in the T-helper 17/regulatory T-cell ratio, the interaction between pathogenic metabolites and immune cells, and shifts in the gastrointestinal microenvironment [12,31]. 

## 3. Pathogenetic Molecular Mechanisms of Osteoarthritis

### 3.1. Main Molecular Mechanisms Involved in Osteoarthritis

The etiology behind OA development encompasses the physiologic onset of cellular aging, a rise in the synthesis of proinflammatory mediators associated with advancing age alterations in epigenetic mechanisms, and a breakdown in mitochondrial function, with all of these leading to a state of heightened inflammation [32].

On a molecular level, maintaining the precise architecture and function of the articular cartilage hinges on Transforming Growth Factor (TGF-β) signaling. Specifically, TGF-β can transmit signals through either the conventional ALK5 type 1 receptor or the ALK1 receptor in chondrocytes [7,33].

Absence of TGF-β signaling in cartilage causes chondrocyte hypertrophy, which then results in the deterioration of cartilage [34]. Moreover, the examination of synovium exhibits the expression of IL6, which is a cytokine independently associated with OA pain and radiographic progression and is secreted by CD34+ and CD90+ fibroblasts [35].

Additionally, genetic alterations, such as those of the Wnt/β-catenin and Ihh signaling pathways, disturb the equilibrium of anabolic and catabolic activity inside the cartilage in joints, thereby triggering permanent extracellular matrix breakdown; they have been identified as the other causative agents of OA [36]. Finally, the locally hypertrophic infrapatellar fat pad participates in cartilage deterioration through the secretion of inflammatory cytokines, adipokines, and growth factors, as well as by responding to the inflammatory local environment in the articulation [37,38].

### 3.2. Main Molecular Mechanisms Involved in Rheumatoid Arthritis

RA is triggered by both genetic and epigenetic variables, as well as pollutants such as cigarette smoke, dust, and the microbiome, which acts as an internal environment [13,39,40]. Moreover, the established association between the HLA-DRB1 locus and RA patients indicates that T-cell selection serves as an essential part in the generation of autoreactive immune responses [41,42].

Dysfunction in cellular immune responses adds to the development of autoantibodies, especially Rheumatoid Factors (RFs) and antibodies against post-translationally modified proteins (such as AMPA and ACPAs), involving antibodies for different alterations such as citrullination, carbamylation, and acetylation, as well as T- and B-lymphocyte infiltration into the synovium [43,44]. In addition to causing cartilage and subchondral bone injury, RA has a notable impact on the composition of synovial fluid, along with the secretory activity of the knee’s infrapatellar fat pad [45].

### 3.3. Main Molecular Mechanisms Involved in Spondyloarthritis

Updated clinical research indicate that mechanical strain has a role in the progression of inflammation and the growth of bones at the enthesis levels [46].

The finding of elevated IL-23 levels in SpA patients validates the participation of the IL-23/IL-17 pathway in SpA development [47].

In addition, SpA is genetically linked with HLA-B*27, which possesses three distinct properties such as peptide-binding selectivity, a misfolding tendency, and a tendency to produce heavy-chain homodimers, which may aid disease etiology [48].

Because misfolded HLA-B*27 components in the Endoplasmic Reticulum (ER) are eliminated by ER-associated degradation (ERAD), either the weak folding or buildup of misfolded HLA-B*27 raise ERAD levels, especially during inflammation [49].

In fact, the unfolding of HLA-B*27 activates transcription factors such as the CCAAT-enhancer-binding protein homologous protein (CHOP) and increases the production of IL-23, INF-β, and IL-1, which are all pro-inflammatory cytokines [50]. Moreover, in rheumatoid synovial tissue and at sites of tissue destruction, such as the cartilage–pannus junction, the majority of pannus cells, especially those invading the cartilage, express pro-inflammatory TNFa [51].

Finally, SpA causes degenerative and atrophic changes to the heel fat pad, which appear to be related to chronic anomalies in the plantar fascia and enthesis [52].

## 4. Outcome Measures for Arthritis

The “Outcome Measures in Rheumatology” (OMERACT) [53] and the “Core Outcome Measures in Effectiveness Trials” (COMET) [54] initiatives are commonly exploited in the scientific literature to define validated outcomes measures for inflammatory conditions of the joints. Some of the most frequently used outcome measures for inflammatory arthritis are hereby reported (Table 1).

### 4.1. Physical Functioning and Disability

#### 4.1.1. HAQ: Health Assessment Questionnaire

This revision specifically targets aspects of physical functioning and disability relevant to those with ankylosing spondylitis. The questionnaire encompasses elements derived from the disability index, addressing areas such as dressing, awakening, eating, walking, hygiene, reaching, grasping, and various daily tasks like using a rear-view mirror, carrying heavy goods, prolonged sitting, and desk work. In total, there are 25 items, with 20 being adapted from the HAQ-DI and 5 being specific to the HAQ-S. The strength of this tool lies in its administration protocol, which can be followed by the patient or by a proxy.

Responses are categorized as follows: 0 = no trouble; 1 = little difficulty; 2 = great difficulty; and 3 = unable to perform. The resulting score range is from 0 to 3 [55].

#### 4.1.2. MHAQ: Modified Health Assessment Questionnaire

The Stanford Health Assessment Questionnaire (HAQ), which was introduced more than 20 years ago, considerably eased the clinical assessment of patients’ capacity to conduct activities of daily living (ADL), and it has since become a generally acknowledged instrument. Both the MHAQ and the HAQ are sensitive to change; however, the HAQ has proven to be more effective in identifying treatment changes than the MHAQ. The scoring techniques differ because the HAQ includes questions about gadgets or help, which may result in a higher final score [56].

#### 4.1.3. AIMS Subscales: Arthritis Impact Measurement Scale

The Arthritis Impact Measurement Scale 2 (AIMS-2) is a questionnaire developed to examine many aspects of the health status of people with arthritis [57]. The questionnaire’s core section contains 57 items classified into 12 scales, which are split into five grades as follows: physical (movement level, walking and bending, hand and finger activity, arm activity, self-care, domestic duties), symptom (arthritic pain), role (job), interpersonal interactions (social activity, family and friends support), and mood (level of tension, attitude) [58].

#### 4.1.4. BASFI: Bath Ankylosing Spondylitis Functional Index

The Bath Ankylosing Spondylitis Functional Index is a self-reported survey designed to describe and track the bodily function of people with ankylosing spondylitis. It consists of eight tasks relating to the patients’ functional anatomy (bending, reaching, shifting position, standing, turning, and mounting steps) and two items measuring the patients’ capacity to deal with daily living.

It consists of 10 items with a numerical answer scale ranging from 0 to 10 and a visual analog scale ranging from 0 cm to 10 cm, which are anchored by adjectival adjectives “easy” and “impossible” [59].

#### 4.1.5. DASH: Disabilities of the Arm, Shoulder, and Hand Outcome Measure

DASH18 is a region-specific evaluation of disability and symptoms for individuals with upper limb musculoskeletal disorders. The items inquire about the degree of trouble in completing different physical tasks over the past seven days due to shoulder, arm, or hand issues (21 items); the degree of intensity of each of the signs of pain, activity-related pain, tingling, weakness, and stiffness (5 items); and (3) the problem’s effect on relationships, work, sleep, and psychological impact (4 items). Each query contains five choices for the responses, ranging from 1 (not difficult to complete, no symptom, or no influence) to 5 (unable to complete, severe symptom, or considerable impact). The responses to the 30 items are combined to provide a raw score, which is then converted into a 0-to-100 scale. A higher score suggests more of a disability [60].

#### 4.1.6. GAT: Grip Ability Test

The GAT is an adaptation of the grip function measure, which is a broad measure of hand function based on daily activities. The GAT is a simple assessment of the function of the hand used for RA patients. During the test, the patients must do three tasks as follows: slip a sock on one hand, place a paper clip on an envelope, and pour water from a jug. The time that it takes to complete the challenges determines the score, with a maximum of 60 s for each assignment. A score below 20 indicates normal operation [61,62,63].

### 4.2. Pain

#### 4.2.1. VAS: Visual Analog Scale

The pain VAS is a graphic scale comprising a horizontal (HVAS) or vertical (VVAS) line that is generally 10 cm long and is supported by two verbal labels, with one for each symptom’s extremity. Guidelines, which report the time limits, and verbal description anchors have varied widely across the literature depending on the scale’s intended use. The VAS for pain is a single-item scale. The pain intensity scale is commonly anchored by “no discomfort” (0 points) and “worst conceivable pain” (100 points). Participants are usually asked to assess current pain within the last day [64].

#### 4.2.2. NRS: Numeric Scale

The NRS for pain is a one-dimensional assessment of the level of pain of patients with rheumatic disease-related chronic pain. It is an 11-point numerical scale, with zero indicating one extreme (no pain) and 10 indicating the opposite extreme (worst pain imaginable). Individuals with chronic pain opt for the NRS over different pain severity scales, such as the pain VAS, since it is more understandable and easier to complete. However, survey results from chronic back pain patients and those with severe hip and knee OA revealed that the pain NRS is inadequate in assessing the convoluted nature of the sensation of pain or improvements resulting from symptom changes [65].

### 4.3. Fatigue and Tiredness

#### MAF: Multidimensional Assessment of Fatigue

The MAF was established in 1991 to assess many aspects of fatigue in individuals with RA. The MAF assesses fatigue according to four categories as follows: severity, distress, impairment with regular daily tasks (chores, food preparation, showering, getting dressed, working, sexual activity, shopping, walking, and training), as well as recurrence and change over the past week. It consists of 15 questions that generate a global score and an additional inquiry (“To what extent has your fatigue changed over the last week?”). The MAF also has applications for various rheumatological disorders, including OA, ankylosing spondylitis, systemic lupus erythematosus, and fibromyalgia [66].

## 5. Diagnosis of Gut Microbiota Dysbiosis and Osteoarthritis

### 5.1. Gut Dysbiosis

Dysbiosis may be triggered by factors specific to the host such as genetic makeup, their state of health, which includes the presence of infections or inflammation, lifestyle, or environmental elements such as hypercaloric and low-fiber nourishment, xenobiotics, and hygiene [11,67].

Improved knowledge of the microbiota of the gut, their metabolites, and links with the host might lead to the development of novel diagnostic and therapeutic techniques [68]. Tissue sampling along with additional invasive techniques of diagnosis are often needed to determine a diagnosis as well as to track disease progression and medication effectiveness. Consequently, non-invasive and reliable markers are required, such as microbiota species and their metabolites, which serve both as prognostic and diagnostic aids [69] (see Table 2). 

Molecular examination of stool specimens is offered as a less-invasive means of detecting patients with gastrointestinal dysbiosis [70]. After the cultures have been collected, several particular indicators are used to detect substantial deviations from the normal bacterial species. Bacterial marker profiling, for example, includes 54 probes focusing on 16S RNA genes (V3–V7) and over 300 bacterial markers. The dysbiosis index score varies between 1 and 5, with 2 representing dysbiosis, and the results of each taxon range from −3 to 3, where the lowest values represent a decreased number compared to those of the reference population [71].

Taxon-based approaches are another legitimate option. Such studies have used relevant taxa to develop dysbiosis indices that only rely on the abundance of certain species [71].

### 5.2. Inflammatory Joint Diseases

The diagnosis of inflammatory joint disease may be achieved through different pathways.

Firstly, the patients are requested to perform serologic tests. In reality, autoantibodies have been employed to inform the diagnosis of many autoimmune illnesses. Serum Rheumatoid Factor (RF) is strongly connected with rheumatoid arthritis and may validate a clinical suspicion, although it is sometimes related to other autoimmune pathologies [6].

Antibodies against Cyclic Citrullinated Peptides (CCPs or ACPAs) represent a reliable diagnostic tool, especially for RA patients [59].

Moreover, by the blood work, the clinician is able to identify increases in acute phase markers that increase during inflammation such as the Erythrocyte Sedimentation Rate (ESR) and C-Reactive Protein (CRP). However, they are not specific and may also increase in cases of infection [72].

Assessment of axial SpA by plain radiographic imaging of the sacroiliac joints can show typical alterations, including erosions, sclerosis, and joint ankyloses [10]. These alterations may not be apparent in early disease. Additionally, individuals may develop non-radiographic axial SpA, with its symptom intensity being similar to that of radiographically visible illnesses.

In patients with a high pretest probability, being HLA-B27-positive increases the chance of SpA. HLA-B27 testing is required for individuals with acute anterior uveitis. For individuals with uveitis, a fast ophthalmology referral and a slit-lamp assessment are needed to confirm the diagnosis and eliminate infectious processes [24].

Finally, the diagnosis is also based on the evaluation of the biomarkers of disease activity. Here, we recognize the Tender Joint Count (TJC). The TJC measures the quantity of inflammatory synovial tissue and is linked with the degree of discomfort and edema [73]. The distal interphalangeal, proximal interphalangeal, and metacarpophalangeal hand joints are examined together with the metatarsal phalangeal and distal interphalangeal joints of the feet and in addition to the shoulder, elbow, wrist, hip, knee, ankle, and tarsus, as well as temporomandibular, sternoclavicular, and acromioclavicular articulations [74].

Another widely utilized biomarker is that of the Rheumatoid Arthritis Disease Activity Index (RADAI), although it is not often employed in routine diagnosis. The RADAI asks about pain in 16 different joint groups. Pain is assessed from 0 to 3, with 0 representing the absence of pain, 1 representing mild pain, 2 representing moderate pain, and 3 representing severe pain, with an overall score ranging from 0–48. The RADAI also comprises three 10 cm VAS scales for the overall activity of the pathology in the preceding six months. Morning stiffness has six choices for responding to that range from 0 (none) to 6 (all day). Scores are reported according to a scale from 0 to 10. The RADAI score is the average of the patient’s replies to many items [73].

Finally, the Ritchie Index (RAI) includes 52 joints, including the shoulder, elbow, wrist, hip, knee, ankle, talocalcaneal, tarsus, and cervical spine, which are evaluated solely for tenderness. The metacarpophalangeal and proximal interphalangeal joints are tested in groups, whereas the temporomandibular, sternoclavicular, and acromioclavicular joints are evaluated collectively. The assessment comprises a grading system of 0 = non-tender, 1 = tender, 2 = painful with wincing, and 3 = tender with withdrawal, with a total score ranging from 0 to 78 [73].

## 6. Therapeutical Options

A number of research investigations have found attainable illness-associated microbial patterns that might be employed to diagnose the early stages of disease, follow progression, and assess treatment effectiveness. The integration of the recognition of microbiota-derived chemicals in urine, blood, or feces may increase both the prognostic and diagnostic use of microbiome profiles.

A detailed understanding of the interactions between the microbiota and their aid will help the evolution of successful therapies based on microbiota. Examples of microbiota manipulation include introducing new beneficial strains or removing unwanted ones, as well as modifying the entire ecosystem by transplanting the fecal microbiota into it. A complementary technique is focused on the usage of microbial metabolites, which includes promoting or inhibiting the production of certain metabolites (Table 3).

### 6.1. Osteoarthritis

The treatment of OA can include non-surgical treatments, such as changes in lifestyle (e.g., encompassing education, self-management, physical activity, weight reduction), pharmacological therapy (paracetamol, topical or oral NSAIDs, intra-articular injections of corticosteroids and hyaluronic acid), nutraceuticals, physical therapy, oxygen–ozone therapy, rehabilitation, and also some new therapies such as mesenchymal stem cell injections, nerve blocks, and platelet-rich plasma injections [75,76,77,78,79]. On the other hand, these actions merely decrease the symptoms of OA in patients, leading inevitably to surgery [80,81].

In this context, to counteract OA and gut dysbiosis, prebiotics and probiotics have proven to be effective [82]. Probiotics can modulate the host’s gastrointestinal microbiota by encouraging the development of beneficial microorganisms, thereby decreasing OA symptoms, as demonstrated by randomized, double-blind, placebo-controlled studies [83,84,85]. Moreover, Lactobacillus and Bifidobacterium appear to lower inflammatory markers such as that of the C-Reactive Protein [86].

Diet has been demonstrated to provide a substantial influence on the gut flora. Dietary nutrients may alter the gut microbiota by modifying their microenvironment, such as the makeup of their microbiota and metabolism, as well as the host’s immunological responses. Several studies have found a link between increased fiber consumption and a reduction in OA discomfort. Reducing a high-fat diet is a rational treatment approach, but it is unlikely to reverse or fully stop OA development [87]. Recent evidence also showed how vitamin D that has a recognized role for bone health and muscle performance [88,89] can also modulate the microbiota composition leading to an increase in beneficial bacteria (e.g., Ruminococcaceae, Faecalibacterium, etc.) and decreasing in Firmicutes [90].

Antibiotic dosing used to modify the gut microbiota, the restoration of the intestine’s barrier integrity, and exercise are all intriguing study areas. Exercise can maintain the intestine shape and lower systemic inflammation despite the presence of a high-fat diet, thereby indicating that physical activity exhibits distinct gut microbiota that are not related to food [91].

Fecal microbiota transplantation (FMT) implies the transfer of feces from a healthy donor into a recipient’s distal gastrointestinal tract in order to cure gut microbiota-related disorders. It has been proven that the microbiota of the gut from varied cohorts of people can impact the degenerative course of joint injury-induced OA in germ-free mice, thereby indicating an exciting prospect for the utilization of FMT in the medical management of OA [92].

### 6.2. Rheumatoid Arthritis

Research literature focuses on the direct immunological repercussions of dysbiosis, which has already revealed some new therapy prospects, such as those of maintaining and restoring a functioning gut barrier or of inhibiting the migration of gut-primed immune cells out of the intestines [93]. These therapy options also provide an opportunity to intervene early in the disease cycle, potentially reducing RA symptoms and the need for lifetime pharmaceutical medications. Thus, the notion that existing successful RA medications might also affect the intestinal bacterial flora increases the prospect that gut microbiota modification could be exploited for therapeutic reasons for RA patients [93].

Recent evidence has been carried out to examine the impact of probiotics on disease activity and cytokine profiles; indeed, a meta-analysis of four similar trials found no evidence of the benefit of probiotics as an additional treatment for RA [94]. However, given the sample sizes and study designs available, this conclusion cannot yet be deemed definitive. In fact, another review concluded that RA patients, who often exhibit inflammation in the intestinal wall resulting in a heightened intestinal motility that allows certain bacteria to breach the gut barrier and induce infection, can use probiotics to mitigate inflammation [94].

Moreover, Mediterranean, and vegetarian diets have been demonstrated to reduce disease activity among the dietary therapies evaluated for RA to date [95].

Patients with slowly progressing or severe RA may benefit from the injection of low-dose oral corticosteroids while awaiting DMARD treatments (hydroxychloroquine [HCQ], MTX, or sulfasalazine [SSZ]) to have an effect [72].

Regardless of the improved application of traditional synthetic DMARDs (csDMARDs) and the increasing number of accepted biologic DMARDs (bDMARDs) for the treatment of RA, such as five TNF inhibitors, two IL-6 inhibitors, an IL-1 inhibitor, two anti-CD20 mAbs, and a CTLA4 inhibitor, an evident, unresolved need stands in the management of RA patients as recovery can be obtained in less than half of patients [96,97]. In 1991, Jak inhibitors were introduced, and three drugs are currently approved as follows: tofacitinib, baricitinib, and Upadacitinib. These drugs have been found to be more clinically effective than methotrexate in early RA [98,99].

Finally, surgery represents a valid option for some of the patients. The knee is the most often afflicted joint in individuals with OA and RA, and approximately 25% of RA patients require a knee prosthesis within 22 years [100]. Total Knee Arthroplasty (TKA) achieves better outcomes in RA patients younger than 60 years [101].

Regarding the shoulder joint, between the 1970s and 1990s, the surgical therapy used for individuals with RA who required an arthroplasty was either a hemiarthroplasty (HA) or a Total Shoulder Arthroplasty (TSA). With the introduction of Reverse Shoulder Arthroplasty (RSA) [5], arthritic individuals with pain and significant shoulder impairment began to exhibit a reduction in shoulder discomfort and a restoration in arm mobility without a functional rotator cuff [102].

### 6.3. Spondyloarthritis

The first-line therapy of SpA is represented by NSAIDs and lifestyle modifications [103,104,105]. The latter typically consists of physical aids, such as physical support devices and biomechanical interventions.

Concurrently, patients are asked to reduce the modifiable risk factors by weight loss through physical exercise, rest, and cryotherapy. Physiotherapy aims at preventing muscular atrophy and joint stiffness [103].

Anti-TNF medications are the gold standard for the treatment of SpA-IBD patients. Since 2000, TNF-α inhibitors have been utilized to control SpA. TNF-α inhibitors are often used after tDMARDs have failed [106]. Currently, five biological TNF-α-specific drugs are available such as adalimumab (ADA), golimumab (GOL), infliximab (IFX), etanercept (ETN), and certolizumab pegol (CTZ) [75]. Moreover, additional therapeutic options can be employed, such as those of IL-17 inhibitors and JAK inhibitors [107].

Intra-articular steroid injections into the afflicted joints can be mainly utilized in the management of peripheral symptoms and in the treatment of active sacroiliitis in the axial subset, but they only offer transient relief [104].

Orthopaedic surgery for SpA, particularly surgery that replaces major joints, is a sign of the severity and failure of medical therapy [108]. THA and arthrodesis are less common for individuals under the age of 60, whereas spinal surgery is less common for those under the age of 40 [108].

## 7. Conclusions

In conclusion, accumulating evidence highlights that the gut microbiota could represent an important part not only in metabolic, immune-mediated, and neurological illnesses but also in joint inflammation. Environmental variables, notably poor diets, and drugs, have the most harmful impact on the gut flora. A dysbiotic microbiota can disrupt the intestinal mucosal barrier, causing chemicals from the food and bacteria to flood tissues and organs, thereby severely impacting the host’s immune system and metabolism. At the heart of this notion is the disarrangement and dysbiosis of physiologic gut homeostasis, which is followed by both adaptive and innate immune responses caused by the migration of bacteria and bacterial metabolites into the bloodstream and into the joint. This cycle eventually ends in low-grade inflammation in the joint, which takes part in the development of inflammatory joint disease.

## 8. Future Perspectives

In upcoming years, the intersection of microbiome research and inflammatory joint disorders is expected to bring up new paths for clinical intervention and therapeutic progress. From a clinical point of view, future studies should aim to investigate innovative microbiota-targeted treatments achieved by supplementing prebiotics and probiotics, which include Bifidobacterium and Lactobacillus, components of the gut microbiota, and their effects on joint pathology, as well as by developing protocols to predict the onset of inflammatory joint disease based on gut dysbiosis. Furthermore, potential areas of study, such as in-depth studies on the host gene regulation that is related to the gut–joint axis, and the relevance of precise cellular causative agents to the gut microbiota are expected.

## Figures and Tables

**Table 1:** Outcome measures and classifications.

Outcome Measures and Classifications	
Physical functioning and disability	HAQ
	MHAQ
	AIMS
	BASFI
	DASH
	GAT
Pain	VAS
	NRS
Fatigue	MAF

HAQ: Health Assessment Questionnaire; MHAQ: Modified Health Assessment Questionnaire; AIMS subscales: Arthritis Impact Measurement Scale; BASFI: Bath Ankylosing Spondylitis Functional Index; DASH: Disabilities of the Arm, Shoulder, and Hand outcome measure; GAT: Grip Ability Test; VAS: visual analog scale; NRS: Numeric Scale; MAF: Multidimensional Assessment of Fatigue.

**Table 2:** Diagnosis.

Diagnosis	
Gut dysbiosis	Fecal analysis
	Biopsy
Inflammatory joint disease	Serologic tests (anti-RF and anti-CCP; ESR and CRP)
	X-ray
	Genetic testing (HLA-B27)
	Disease activity biomarkers (TJC, RADAI, RAI)

RF: Rheumatoid Factor; CCP: Cyclic Citrullinated Peptide; ESR: Erythrocyte Sedimentation Rate; CRP: C-Reactive Protein; TJC: Tender Joint Count, RADAI: Rheumatoid Arthritis Disease Activity Index; RAI: Ritchie Index.

**Table 3:** Diseases and treatment measures.

Treatment	
Osteoarthritis	Lifestyle modifications
	Probiotics
	Antibiotics
	FMT
	DMOADs
	Prosthesis implant
Rheumatoid arthritis	Lifestyle modifications
	Probiotics
	Corticosteroids
	DMARDs
	Prosthesis implant
Spondylarthritis	Lifestyle modifications
	NSAIDs
	DMARDs
	Anti-TNF
	Prosthesis implant

FMT: Fecal microbiota transplantation; DMOADs: Disease-Modifying Osteoarthritis drugs; NSAIDs: Non-Steroidal Anti-Inflammatory Drugs; DMARDs: Disease-Modifying Anti-Rheumatic Drugs; TNF: Tumor Necrosis Factor.

## Data Availability

Not applicable.
